# Feasibility study of individualized optimal positioning selection for left‐sided whole breast radiotherapy: DIBH or prone

**DOI:** 10.1002/acm2.12283

**Published:** 2018-02-13

**Authors:** Hui Lin, Tianyu Liu, Chengyu Shi, Saskia Petillion, Isabelle Kindts, Caroline Weltens, Tom Depuydt, Yulin Song, Ziad Saleh, Xie George Xu, Xiaoli Tang

**Affiliations:** ^1^ Nuclear Engineering and Engineering Physics Rensselaer Polytechnic Institute Troy USA; ^2^ Department of Medical Physics Memorial Sloan‐Kettering Cancer Center New York USA; ^3^ Department of Radiation Oncology University Hospitals of Leuven Leuven Belgium

**Keywords:** DIBH, Prone, machine learning, breast cancer, OAR sparing

## Abstract

The deep inspiration breath hold (DIBH) and prone (P) position are two common heart‐sparing techniques for external‐beam radiation treatment of left‐sided breast cancer patients. Clinicians select the position that is deemed to be better for tissue sparing based on their experience. This approach, however, is not always optimum and consistent. In response to this, we develop a quantitative tool that predicts the optimal positioning for the sake of organs at risk (OAR) sparing. Sixteen left‐sided breast cancer patients were considered in the study, each received CT scans in the supine free breathing, supine DIBH, and prone positions. Treatment plans were generated for all positions. A patient was classified as DIBH or P using two different criteria: if that position yielded (1) lower heart dose, or (2) lower weighted OAR dose. Ten anatomical features were extracted from each patient's data, followed by the principal component analysis. Sequential forward feature selection was implemented to identify features that give the best classification performance. Nine statistical models were then applied to predict the optimal positioning and were evaluated using stratified k‐fold cross‐validation, predictive accuracy and receiver operating characteristic (AUROC). For heart toxicity‐based classification, the support vector machine with radial basis function kernel yielded the highest accuracy (0.88) and AUROC (0.80). For OAR overall toxicities‐based classification, the quadratic discriminant analysis achieved the highest accuracy (0.90) and AUROC (0.84). For heart toxicity‐based classification, Breast volume and the distance between Heart and Breast were the most frequently selected features. For OAR overall toxicities‐based classification, Heart volume, Breast volume and the distance between ipsilateral lung and breast were frequently selected. Given the patient data considered in this study, the proposed statistical model is feasible to provide predictions for DIBH and prone position selection as well as indicate important clinical features that affect the position selection.

## 1 INTRODUCTION

Breast cancer is the most common malignant disease in women in the United States, second to the lung cancer as the leading cause of cancer death.^1^ While the whole breast irradiation (WBI) has demonstrated a significant overall survival benefit and low recurrence rate,^2^, ^3^ studies have shown the increased risk of cardiac and lung disease associated with the WBI.^4^

**Figure 1:**
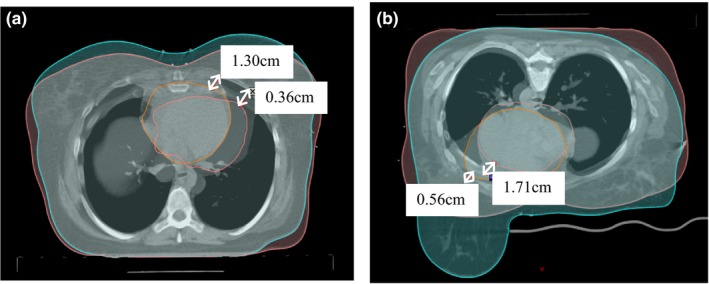
(a) Image fusion of the CT scans in the Free Supine versus the DIBH position. The supine position is in pink, and the DIBH position is in blue. For the supine position, the heart‐to‐chest distance was 0.36 cm, and this distance increased to 1.30 cm when the patient was positioned in DIBH. (b) Image fusion of the CT scans in the Free Supine versus the prone position. The supine position is in pink, and the prone position is in blue. For the supine position, the heart‐to‐chest distance was 1.71 cm, and this distance decreased to 0.56 cm when the patient was positioned in prone. The supine scan was rotated 180 degrees to align with the prone scan.

The deep inspiration breath hold (DIBH) is one common heart sparing irradiation technique for left‐sided breast patients. Since the heart can be displaced away from the left breast during deep inspiration in most patients, one approach to reducing incidental cardiac irradiation is to treat patients during this portion of the respiratory cycle; i.e., using DIBH. Shown in Figure 1a of an image fusion, the distance between the chest wall and heart of the patient increased from 0.36 cm to 1.30 cm from supine free breathing (FB) to DIBH. On the other hand, the ipsilateral lung involvement might be increased due to the deep breath hold.

Prone (P) position is another heart sparing technique. While the prone position can dramatically reduce the lung dose, reduction in the heart exposure is controversial.^5^, ^6^ The image fusion (Figure 1b) indicated that the heart was situated at further distances from the chest wall in the supine position (heart‐to‐chest distance equals to 1.71 cm), whereas it lays more adjacent to the chest wall in the prone position (heart‐to‐chest distance decreased to 0.56 cm).

Currently, for patients suitable for both techniques, clinicians select one technique that might result in better organs at risk (OAR) sparing. This decision is mainly based on experience, and might not always yield the lowest dose. Our study aims to provide predictions and quantitative guidelines for this clinical decision. Nine statistical learning algorithms are investigated. To evaluate the performance, the prediction results obtained by the models were compared to the ground truth results that have been selected for these trial patients by physicists based on treatment planning.

## 2 MATERIALS AND METHODS

### 2.A Proposed procedures

**Figure 2:**
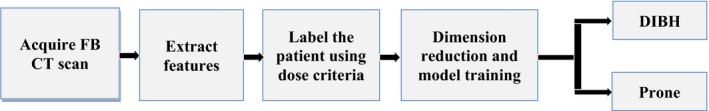
The diagram of model building and prediction process.

Figure 2 is the diagram of our proposed classification/prediction training procedure. The supine free‐breathing computed tomography (CT) scans of the patients were used as input for model training. The first step is to extract anatomical features. Ten clinically relevant features were extracted. The next step is to label the patient to different classes based on user‐defined dose criteria. In this case, there are two classes — DIBH and prone position. Then we apply dimension reduction techniques such as the principle component analysis (PCA) or feature selection to these features and train the model upon that. After several rounds of evaluation, the model is built.

When a new patient FB CT comes in, the same ten features would be extracted and employed as the input of the pre‐trained model to predict which class the patient should belong to. We say that the predicted class is the optimum position for that patient. Each of these steps will be described in detail in the following sections.

### 2.B Patient data and planning

Sixteen left‐sided breast cancer patients were included in the prospective trial conducted by Department of Radiation‐Oncology, University Hospitals of Leuven, Belgium at the time adjuvant WBI was planned after lumpectomy. Patients then received three noncontrast CT in the following different positions during the simulation procedure: (1) standard supine position in FB; (2) supine position with gating in DIBH; and (3) prone position. The detailed procedures were described by Verhoeven et al.^7^ The CT data were then transferred to the treatment planning system (Eclipse; Varian Medical Systems) for delineation and planning. Target breast volumes and OAR (lungs, heart, left anterior descending artery and contralateral breast [CLB]) were delineated. The delineations of the CT scans in supine and prone position were done by the same radiation oncologist.

Standard tangent fields with compensator design^8^ were used for WBI to improve dose homogeneity. Typical WBI prescription (200 cGy × 25)was used. The normalization point was placed at lung‐chest wall interface anterior of the rib. For each patient in each position, the plan that best covered the whole breast PTV (optimized not to exceed 110%) and minimized the OAR doses (the volume of the heart receiving 25 Gy dose ≤5%, and the volume of ipsilateral lung receiving 20 Gy dose ≤20%) was selected as the optimal treatment plan. The dose distributions were reviewed in three dimensions. Isodose distributions and dose volume histograms were used to analyze whole breast PTV coverage, dose homogeneity, and doses to OAR. To evaluate the doses to OAR, mean doses, V_25_ heart, V_20_ ipsilateral lung, and V_5_ CLB were analyzed.

Treatment plans were generated for all the three positions of the patient data according to our clinical guidelines. By comparing three treatment plans of each patient, the position (Supine‐free or DIBH or prone) that introduces least OAR doses was selected as the patient label. In this study, we investigated heart toxicity‐based criteria and weighted OAR toxicities‐based criteria. OAR includes heart, ipsilateral lung and CLB, and the weighted toxicity was defined as 0.6 × V_25_ heart + 0.3 × V_20_ lung + 0.1 × V_5_ CLB. Different weights were assigned to the OARs to reflect the relative significance of OAR during the left‐sided breast treatment: the heart is given the highest weight, the ipsilateral lung is the second, and then the CLB.

### 2.C Features extraction and data preprocessing

To train the classifier and predict the optimal position, we extracted the anatomical features from the CT scan. Since each patient would have an FB scan, the feature extraction is done from the FB scans. The following 10 clinically relevant features are extracted and used as the input for the statistical models, and the mean and standard deviation of each feature value are reported (See Table 1).

**Table 1:** Mean and standard deviation value of each feature derived from the patient supine free breathing CT scans

Features (from FB scans)	Mean ± SD
Breast volume (cm^3^)	575 ± 299
Heart volume (cm^3^)	467 ± 57
Ipsilung volume (cm^3^)	1230 ± 224
Distance between heart and breast (cm)	10.4 ± 3.7
Distance between ipsilung and breast (cm)	10.0 ± 2.1
In‐field heart volume (cm^3^)	10.89 ± 3.74
In‐field ipsilung volume (cm^3^)	154.56 ± 58.01
Laterality of heart (cm)	6.9 ± 1.1
Ratio of heart volume to ipsilateral lung volume	0.39 ± 0.09
Breath‐hold motion (cm)	1.3 ± 0.4

#### 2.C.1 Volumes of the breast, heart, and ipsilateral lung

Breast volume has long been used as an important indicator in selecting the optimal positioning for whole breast treatment.^9^, ^10^, ^11^ Heart and ipsilateral lung volume were also selected — the larger the heart and ipsilateral lung volume are, the more likely they would be irradiated.

#### 2.C.2 The distance between heart and breast, and the distance between ipsilateral lung and breast

The distance between OAR (heart and ipsilateral lung) and breast is defined as the distance between the mass centers of OAR and the PTV Breast. All distances were automatically extracted from all patients using CERR.^12^ These two distance features are important because both DIBH and prone positioning can cause a demonstrable OAR shift, which in some cases, would compromise optimal OAR sparing.

#### 2.C.3 In‐field heart and ipsilateral lung volumes

**Figure 3:**
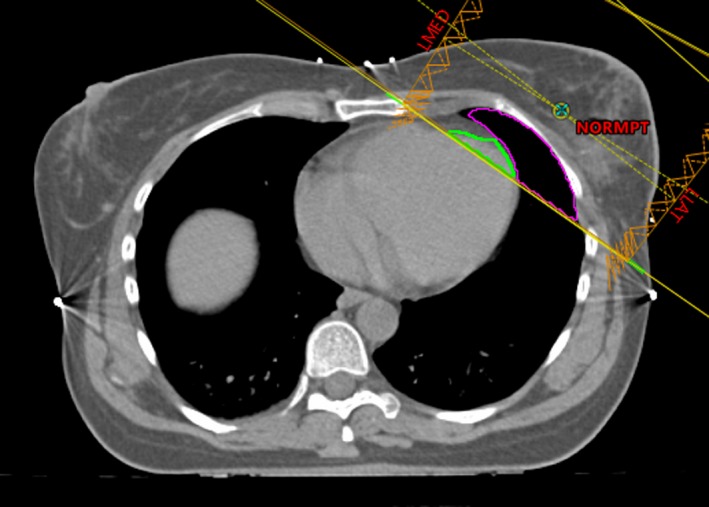
An illustration of the lung and heart volumes in the treatment field. The green contour is the amount of the heart in the field, and magenta is the amount of the ipsilateral lung in the field.

These two features are self‐explanatory by the names. Figure 3 is an example.

#### 2.C.4 Laterality of the heart

**Figure 4:**
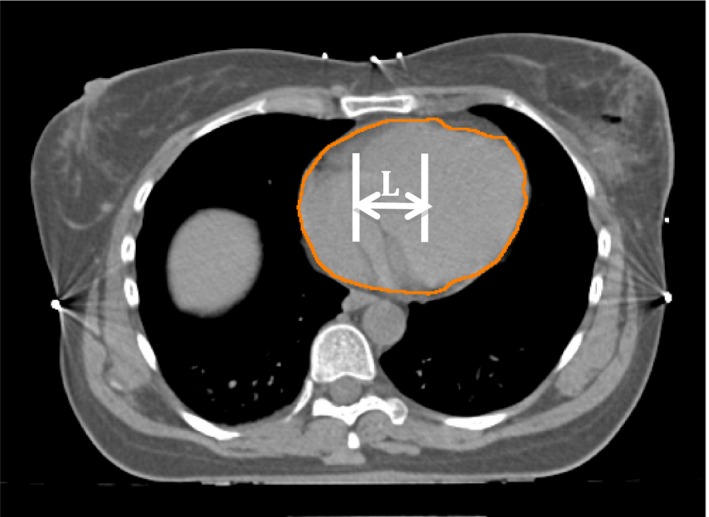
Illustration of the laterality (L in the figure) of the heart to the chest wall.

As shown in Figure 4, the laterality of the heart is defined as the distance between the center of the heart and the center of the chest along the right‐to‐left direction. The further away the heart is from the center of the chest, the more likely it will be in the tangent fields.

#### 2.C.5 The ratio of heart volume to ipsilateral lung volume

Inspired by Zhao et al.,^13^ this feature was chosen to address the concern that when both heart and lung volumes are large, the heart volume alone might not be an effective feature, so we need to normalize the heart volume to ipsilateral lung volume.

#### 2.C.6 Breath‐hold motion

**Figure 5:**
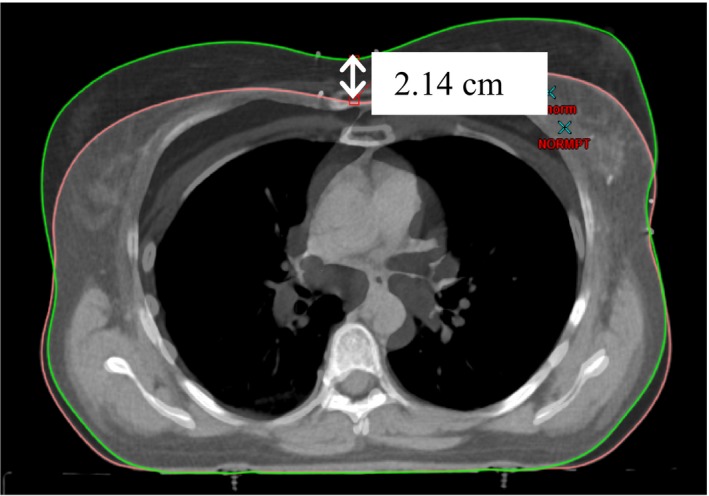
An illustration of the breath hold motion between free breathing and DIBH position of a patient. The pink body contour is FB, and the green is DIBH.

As shown in Figure 5, when the patient took a breath hold, the motion of the anterior chest was 2.14 cm. Usually, this feature is correlated with how much the heart is being moved away from the chest wall.

### 2.D Statistical learning algorithms

The following nine statistical learning algorithms were used to develop the predictive models: nearest neighbors, support vector machine (SVM) with linear and radial basis function (RBF) kernel, Decision Tree, Random Forest, AdaBoost, Naive Bayes, linear and quadratic discriminant analysis (QDA).

#### 2.D.1 Nearest neighbors classification

The principle behind nearest neighbor methods is to find a predefined number. A most common way of metric measurement is Standard Euclidean distance. Nearest Neighbors is often successful in classification situations where the decision boundary is irregular. In this current analysis, the classification based on the k nearest neighbors of each query point was implemented, where *k* is an integer value specified by the user.

#### 2.D.2 Support vector machine

SVM searches for the linear hyper‐plane that can separate binary classes optimally. The optimized hyper‐plane is the one that produces the maximal margin between two classes. Given training vectors in two classes *x*_*i*_ **∈ ℜ^*p*^, where *i* = 1,2,…,*n*, and a vector *y *∈ {1, −1}^*n*^, SVM solves the following problem^14^:

(1) $$\min_{w,b,\zeta }\frac{1}{2}w^{T}w+C\sum_{i=1}^{n}\zeta_{i}$$

Subject to

(2) $$y_{i}\left(w^{T}\phi \left(x_{i}\right)+b\right)\geq 1-\zeta_{i},\zeta_{i}\geq 0,i=1,2,\cdots ,n$$

The SVM model can be applied to both linearly and nonlinearly separable data. For nonlinearly separable data, the SVM first maps the data with a kernel function and then searches for a linear optimally separating hyper‐plane in the new space. Prediction is made according to which side of the hyper‐plane the subject lies on. In this study, the SVM was implemented with a linear and RBF kernel.

#### 2.D.3 Decision tree

Decision Trees predicts the value of a target variable by learning simple decision rules inferred from the data features. Input data are split into two or more subgroups according to the best split in input variables. The splitting continues until stop conditions are met. For training data, given training vectors *x*_*i*_ ∈ ℜ^*p*^, where *i* = 1, 2, …, *l* and a class vector *y* ∈ ℜ^*l*^, a decision tree is built using recursive partitioning algorithm such that the samples with the same labels are grouped.^14^ For each candidate split *θ* = (*j*, *t*_*m*_) consisting of feature *j* and threshold *t*_*m*_, the data *Q* at the node is split into *Q*_*l*_(θ) and *Q*_*r*_(θ), where

(3) $$Q_{l}\left(\theta \right)=\left(x,y\right)\left|x_{i}\leq t_{m}\right.$$

(4) $$Q_{r}\left(\theta \right)=Q\backslash Q_{l}\left(\theta \right)$$

The impurity at the node can be evaluated by using an impurity function *H*. One of the typical choices is called Cross‐Entropy, where *H* is defined as

(5) $$H\left(x_{m}\right)=-\sum_{k}p_{\mathrm{mk}}\log\left(p_{\mathrm{mk}}\right)$$

*m* refers to the current node, and *p*_*mk*_ are fractions that represent the percentage of each class shown in the child node that results from a split in the tree.

#### 2.D.4 Random forest

In random forests, multiple trees are built to classify an object based on features. A sample of training set taken at random but with replacement is used to build a tree. When growing the tree, the best split is chosen among a random subset of the input features. As a result of this randomness, the model selects the classification/regression results that get the most votes from trees in the forest, and thus help reduce the variance of the final model.

#### 2.D.5 AdaBoost

An AdaBoost classifier is an ensemble technique that fits a classifier on the training data and then creates a second model which attempts to correct the weights of incorrectly classified instances. The core principle of AdaBoost is to utilize multiple weak classifiers on repeatedly modified versions of the data so that a strong classifier can finally be generated.

#### 2.D.6 Naive Bayes

Given a class variable *y* and a dependent feature vector *x*_1_ through *x*_*n*_, Bayes' theorem states the following relationship^14^:

(6) $$P\left(y\left|x_{1},\cdots ,\right.x_{n}\right)=\frac{P\left(y\right)P\left(x_{1},\cdots ,x_{n}\left|y\right.\right)}{P(x_{1},\cdots ,x_{n})}$$

The major difference of different naive Bayes classifiers is the assumptions they make regarding the distribution of *P*(*x*_i_|*y*). The Naive Bayes classifier used in this study is Gaussian Naive Bayes, where the likelihood of the features is assumed to be Gaussian:

(7) $$P\left(x_{i}\left|y\right.\right)=\frac{1}{\sqrt{2\pi \sigma_{y}^{2}}}e^{-\frac{{\left(x_{i}-\mu_{y}\right)}^{2}}{2\sigma_{y}^{2}}}$$

#### 2.D.7 Discriminant analysis

The linear/quadratic decision boundary of the classifier is generated by fitting class conditional densities to the data using Bayes' rule. Assuming all classes share the same covariance matrix, the model fits a Gaussian density to each class.

### 2.E Dimension reduction and feature selection

Due to the limited training and testing dataset, dimension reduction technique was employed to reduce the data dimensionality while retaining most variance of the data. PCA is a statistical procedure that transforms the original n coordinates of the dataset into a new set of *m* (*m* < *n*) coordinates through linear combination. After transformation, the first principal component accounts for the largest variance and each succeeding principal component accounts for the highest possible variance if it is orthogonal to the preceding components. Since PCA is sensitive to the relative scaling of the original data, data normalization needs to be applied before PCA.

Feature selection is a process of automatically removing unnecessary features and selecting a subset of features to be used in the predictive modeling. In this paper, we applied sequential forward feature selection (SFFS) algorithm, which employs greedy search to reduce the original n features to a subset of m features where *m *< *n*.^15^ Given the whole *n*‐dimensional features as input

(8) $$F=\{f_{1},f_{2},L,\cdots ,f_{n}\}$$

And the output feature is defined as *Y*_*m*_, where

(9) $$Y_{m}=\left\{y_{i}\left|i=1,2,L\cdots ,m\right.;y_{i}\in F\right\},m=\left(0,1,2,L\cdots ,n\right)$$

SFFS firstly initializes *Y*_*m*_ with an empty subset so that *Y*_0_ = {*φ*}. Then it adds an additional feature *y* ^+^ which can maximize the criterion function to the feature subset, where

(10) $$y^{+}=\arg\max J\left(y_{m}+y\right),\text{where}y\in F-Y_{m}$$

(11) $$Y_{m+1}=Y_{m}+y^{+}$$

This procedure is repeated until the termination criterion is satisfied. In SFFS, the terminal criterion is set as *m* = *p*, where *p* is the number of desired features that we specified a priori. In this study, we set *p* = *n* so that the SFFS will go through all the features and select the feature combination that can generate the best performance. The best feature combination was discovered by iterating forwardly from the first feature to the last, determining which feature combination achieved the best performance during 5‐fold cross‐validation.

### 2.F Model comparison and evaluation

In this study, k‐fold stratified cross‐validation was used to test the model performance as well as picking up the optimal hyper‐parameters. For small training data size, stratified k‐fold cross‐validation is a widely accepted technique to evaluate the generalization capability of a model. The whole dataset is partitioned into *k* smaller subsets, where each subset contains approximately the same percentage of samples of each target class. Every time, the model is trained with the *k* − 1 folds, while the remaining single fold is used to validate the model. This procedure repeats *k* times and the results are combined to generate an estimation of the model performance. In our experiments, we used *k* = 5 and each experiment was repeated for ten iterations using different random seeds. Prediction accuracy and receiver operating characteristic (AUROC) were used for the final evaluation after cross‐validation. Accuracy is defined as the number of correctly predicted samples divided by the number of total samples in the test data. The AUROC is a common method to assess the power of a statistical learning model as its discrimination threshold is varied across all cut‐off values. AUROC takes a value between 0 and 1, with 1 represents a perfect classification prediction, 0.5 represents a classification with discrimination no better than random, and 0 represents a model with all validation instances predicted with a wrong label.

## 3 RESULTS

### 3.A Statistics of the selected features and OAR doses in the treatment plan

The mean and standard deviation of the feature values derived from the patient supine free breathing CT scans are shown in Table 1.

The comparisons of the mean dose to OAR and the mean value of V_25_ heart, V_20_ ipsilateral lung and V_5_ CLB under Supine‐free, DIBH and Prone positioning are summarized in Table 2. Highest mean heart dose and V_25_ heart were observed in supine free position and the dose differences among supine‐free, DIBH and prone positioning were statistically significant. The mean ipsilateral lung dose and the corresponding V_20_ ipsilateral lung are significantly better in the prone position compared to the supine‐free and DIBH. For the CLB, the mean dose and V_5_ CLB were statistically better in two supine positions in comparison with the prone position.

**Table 2:** Mean values of OAR doses and V_25_ heart, V_20_ ipsilateral lung and V_5_ contralateral breast under three positions

Variable	Supine in free breathing	DIBH	Prone
Mean heart dose (cGy)	325.82	194.17	267.21
V_25_ heart (%)	3.76	1.59	3.03
Mean ipsilateral lung dose (cGy)	578.12	597.24	154.34
V_20_ ipsilateral lung (%)	9.04	9.47	2.90
Mean contralateral breast dose (cGy)	27.66	28.33	32.82
V_5_ contralateral breast (%)	0.37	0.39	0.51

### 3.B Performance evaluation of the prediction algorithms after dimension reduction

Initially when we designed the study, we have taken all the three positions into consideration, and the position that introduces least OAR doses was selected as the patient output label. However, after observing the treatment plan results, no patients in this dataset were labeled as supine FB, so in the following model training and validation process, only two classes (DIBH and Prone) exist.

**Figure 6:**
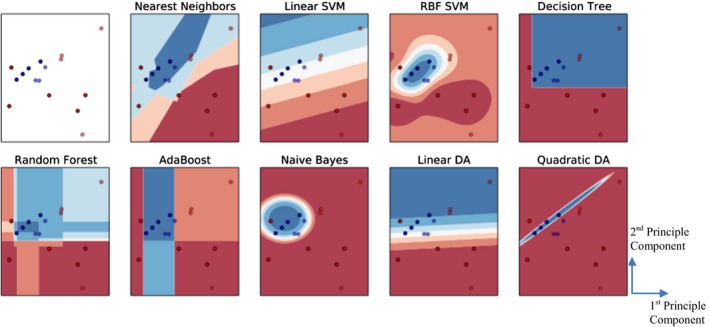
Comparison of different classification algorithms based on heart toxicity. The red dots demonstrate DIBH positioning, and blue dots demonstrate prone positioning. The first subplot (with white background) demonstrates the distribution of the input data, and the blue and red regions in succeeding subplots show the decision boundaries of each model. The first subplot shows the original distribution of the dataset, and the others correspond to the classification boundaries of each model.

**Figure 7:**
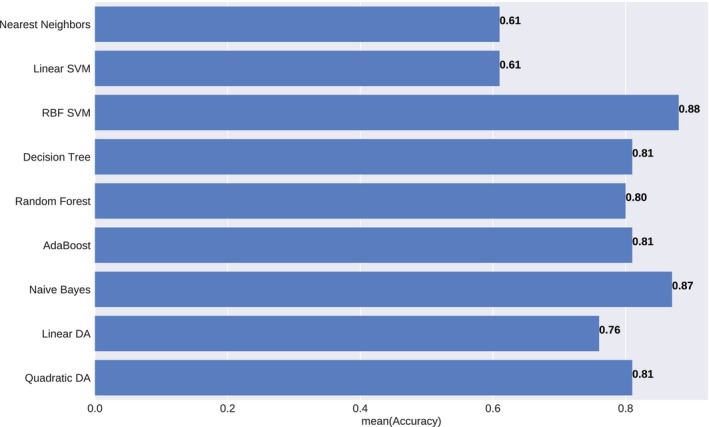
Prediction accuracy of the different statistical model under heart toxicity‐based classification. The results were averaged over ten iterations.

Figure 6 visualized the classification boundaries of all models for the heart toxicity‐based classification. All features were normalized and PCA was applied for dimension reduction. Prediction accuracy and AUROC were utilized to evaluate the model performance. Consequently, SVM with RBF kernel with cost parameter *C *=* *1.0 and γ = 0.6 achieved the highest accuracy – 0.88 and the highest AUROC – 0.80. The comparisons of prediction accuracy of these nine models were demonstrated in Figure 7.

**Figure 8:**
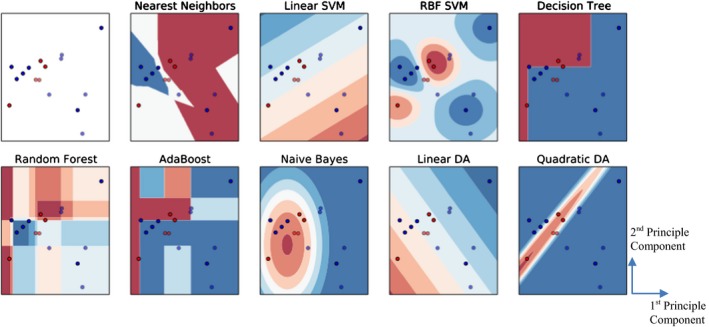
Comparison of different classification algorithms based on all OAR overall toxicity. The red dots demonstrate DIBH positioning, and blue dots demonstrate prone positioning. The first subplot (with white background) demonstrates the distribution of the input data, and the blue and red regions in succeeding subplots show the decision boundaries of each model. The first subplot shows the original distribution of the dataset, and the others correspond to different classification methods.

**Figure 9:**
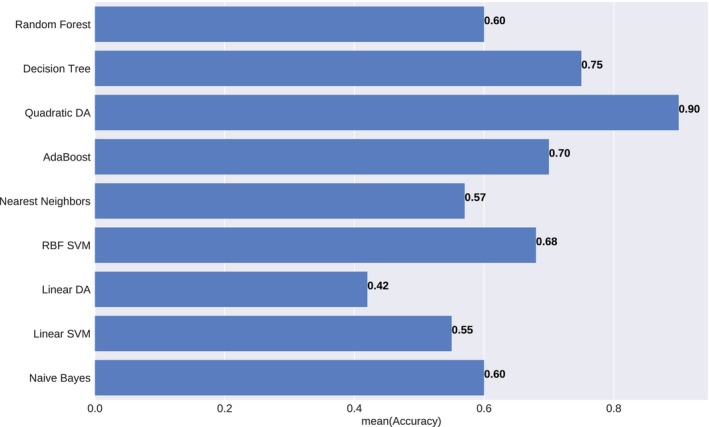
Prediction accuracy of the different statistical model under weighted OAR toxicities‐based classification. The results were averaged over ten iterations.

Figure 8 compared the classification boundaries of all models for the weighted OAR toxicities‐based classification. All features were normalized and PCA was applied for correlation removal. Consequently, QDA achieved the highest accuracy – 0.90 and the highest AUROC – 0.84. The comparisons of prediction accuracy of these nine models were demonstrated in Figure 9.

### 3.C Performance evaluation of the prediction algorithms after sequential forward feature selection

**Figure 10:**
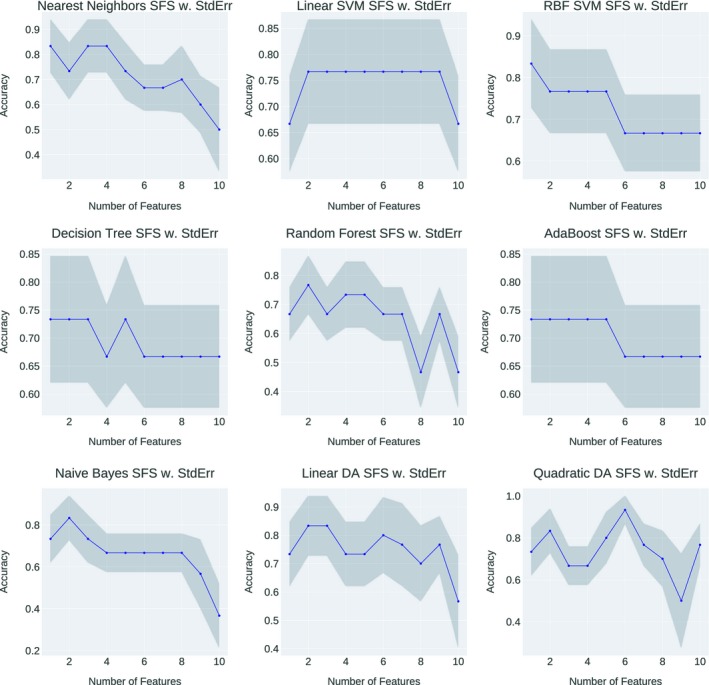
Heart toxicity‐based classification performance variations of each statistical model with different feature combination as the input. The horizontal axis showed the number of features involved in the current training and the vertical axis showed the predictive accuracy.

To provide more insights on what are the causal features should be used to determine the optimal positioning of left‐sided breast treatment, SFFS technique were applied to outweigh the important feature combinations that can generate the best performance. Figure 10 demonstrated the accuracy fluctuation using different feature combinations as the input to the statistical model for heart toxicity‐based classification. The best feature combination of one specific statistical model was conducted when the accuracy arrived the peak value at the *first* time. Although introducing extra features into the model can result in the same accuracy as the first peak, those succeeding features are excluded from the feature selection result, since they cannot prompt the model performance anymore — the model accuracy has already been saturated with the feature combination that leads to the first accuracy peak. Furthermore, using more features with a small dataset would reduce the generalization of the machine learning model, in other words, cause overfitting problem. We have counted the frequency of each feature being selected by the SFFS, and the selected combinations were shown in Table 3.

**Table 3:** The best feature combinations that yield the highest predictive accuracy of statistical models for heart toxicity‐based classification. Features that are consistently selected by all the models are bold

	Nearest neighbors	Linear SVM	RBF SVM	Decision Trees	Random Forest	Ada‐boost	Naive Bayes	Linear DA	Quadratic DA
Selected Features	Vol_B_	Vol_B_	Vol_B_	Vol_B_	Vol_B_	Vol_B_	Vol_B_	Vol_B_	Vol_B_
		Dis_H‐B_			Thickness		Dis_H‐B_	Dis_H‐B_	Vol_H_
									Vol_ipsL_
									Dis_H‐B_
									Laterality
									Thickness
Accuracy	0.83	0.77	0.83	0.73	0.77	0.73	0.83	0.83	0.93

Vol_B_: Breast Volume; Vol_H_: Heart Volume; Vol_ipsL_: IpsiLung Volume; Dis_H‐B_: Distance between Breast and Heart; Thickness: Deep breath motion thickness variation.

Upon feature selection, the model that yields the best predictive accuracy for heart toxicity‐based classification is QDA, where the accuracy is 0.93. By counting the occurrences of each feature, we can observe that for heart toxicity‐based classification, Breast volume was accounted in the best feature combination of every statistical model. The succeeding feature that frequently appeared in the best feature combination was the distance between Heart and Breast. These two features, breast volume and distance between Heart and Breast, were suggested as important indicators for heart toxicity‐based optimal treatment position selection by our study.

**Figure 11:**
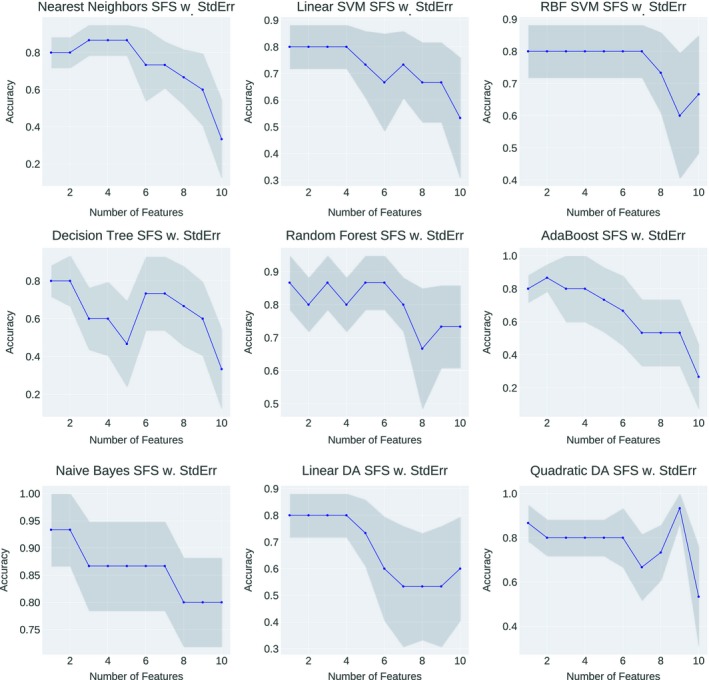
OAR overall toxicities‐based classification performance variations of each statistical model with different feature combination as the input. The horizontal axis showed the number of features involved in the current training and the vertical axis showed the predictive accuracy.

Figure 11 demonstrated the accuracy fluctuation using different feature combinations as the input to the statistical model for OAR overall toxicities‐based classification. The best feature combination of one specific statistical model was conducted when the accuracy arrived the peak value at the first time, and the selected combinations were shown in Table 4.

**Table 4:** The best feature combinations that yield the highest predictive accuracy of statistical models for OAR overall toxicities‐based classification. Features that are consistently selected by all the models are bold

	Nearest neighbors	Linear SVM	RBF SVM	Decision Tree	Random Forest	Ada‐boost	Naive Bayes	Linear DA	Quadratic DA
Selected features	Vol_H_	Vol_B_	Vol_H_	Dis_L‐B_	Vol_B_	Vol_H_	Vol_B_	Vol_H_	Vol_B_
	Vol_ipsL_					Dis_L‐B_			Vol_H_
	Dis_L‐B_								Vol_ipsL_
									Dis_H‐B_
									Dis_L‐B_
									Laterality
									Thickness
									Vol_H_/Vol_L_
Accuracy	0.87	0.80	0.80	0.80	0.87	0.87	0.93	0.80	0.93

Vol_B_: Breast Volume; Vol_H_: Heart Volume; Vol_ipsL_: IpsiLung Volume; Dis_H‐B_: Distance between Breast and Heart; Dis_L‐B_: Distance between Breast and ipsilateral Lung; Vol_H‐in‐field_: Volume of heart in the treatment field; Thickness: Deep breath motion thickness variation; Vol_H_/Vol_L_: Rate of heart volume to lung volume.

Upon feature selection, the model that yields the best predictive accuracy for OAR overall toxicities‐based classification is Naive Bayes and QDA, where the accuracy is 0.93. By counting the occurrences of each feature, we can observe that for OAR overall toxicities‐based classification, the three most frequently selected features are: the volume of heart (5 times), the volume of breast (4 times) and the distance between lung and breast (4 times). Thus, the three selected features above were suggested as important indicators for OAR overall toxicity‐based optimal treatment position selection.

## 4 DISCUSSION

Several studies using statistical learning models in the prediction of optimal positioning in breast cancer treatment have been published.^16^, ^17^, ^18^ Compared to these studies which have taken the supine FB and prone free breathing positions into consideration, our study is the first feasibility study that predicts optimal positioning between DIBH and Prone positions and indicates important features for the sake of OAR sparing. DIBH is a position that can efficiently reduce the cardiac dose for breast radiation therapy,^19^, ^20^, ^21^ and many centers have introduced DIBH to the clinic recently. Our study is timely, as it provides some quantitative clinical guidance to select between DIBH and Prone positions.

We have applied different dose criteria, heart toxicity, and weighted OAR toxicities, to determine the patient positioning label. As shown in Figure 6, if heart toxicity was the only factor influencing the decision, more patients are found to be classified as DIBH‐treated rather than prone‐treated. This is consistent with many previous clinical studies, showing that DIBH is beneficial to heart dose reduction during left‐sided breast treatment. However, if the weighted OAR toxicities (dose to heart, ipsilateral lung, and CLB) are the decision factor, the classification result is the opposite. We believe the reason for this is that the dose to the ipsilateral lung is significantly lower in the prone position compared to the DIBH.^5^, ^22^ By using our model, clinicians can also assign their self‐defined weighting factors to OAR which in turn can address their specific clinical interest or need. In our current study, the largest weighting factor was assigned to the heart, followed by the ipsilateral lung, and the least to the CLB to align with the clinical practice of our institution. These weighting factors can be further optimized and may reflect prediction results.

The limited size of available data remains an obstacle to machine learning in the medical domain. Several studies have investigated the principles to decide the data size required for predictive performance,^23^, ^24^, ^25^, ^26^ but in practice, the amount of data required for machine learning model training depends on many factors, including (1) the complexity of the problem, nominally the number of input features and the complexity of the unknown function that fits input data to the output, (2) the complexity of the machine learning model, nominally the extent of the model's nonlinearity and number of parameters need to be tuned within the model, (3) techniques used to preprocess the data, such as dimension reduction methods and data augmentation. All these factors are problem or data‐specific, so there is really no one‐for‐all rule can simply tell how large the data size is sufficient for a specific machine learning problem. In this study, to address the limited data issue, we have employed dimension reduction techniques including PCA and forward feature selection to extract essential properties from the data and in the meantime, reduce the dimensionality of the input to the machine learning models. After applying PCA, we have found that the first two principal components can describe nearly 90% of the variance in the dataset (the first principal component represented 61% of the variance). This clear pattern can also be recognized through Figures 6 and 8, where class DIBH and class Prone have demonstrated a clustering mode after PCA. From the aspect of model selection, we are also cautious not picking up machine learning models with high complexities, since although a complex model may depict the nonlinearity of the data better, it may also introduce higher risks of overfitting with a limited dataset.

Upon PCA, SVM with RBF kernel and QDA are most likely to be the best‐performing models for the prediction of left‐sided breast treatment optimal positioning. Models like linear SVM, linear discriminant analysis (LDA) and Decision Tree can provide clear and reasonably unambiguous hyper‐plane but failed to improve prediction accuracy substantially, mainly because of their inadequacy to deal with nonlinear inputs as shown in our data. Random forest, which was considered as a superior machine learning model in the context of some predictive studies in radiotherapy,^27^ only provided modest performance in this study.

Compared to PCA, feature selection can provide more insights into causal features that affect the selection of optimal positioning. Some previous studies^13^, ^28^ have shown that forward feature selection can narrow down the input features while achieving better results than applying the entire features set. The same phenomenon was also demonstrated in our experiment, where in Figures 10 and 11, for all the statistical models, it can be observed that the predictive accuracy firstly increased, reached the peak performance and then decreases when more features were added. Some models even can generate fair predictions by relying on only one feature, but this does not mean a single feature is sufficient — as illustrated by the results of QDA, the highest predictive performance was still produced by combing multiple features. This suggests that when performing the optimal positioning of left‐sided breast treatment in the clinic, it is beneficial to select multiple features so that their joint contributions may maximize the OAR sparing effect.

## 5 CONCLUSION

This study demonstrates the feasibility of predicting the optimal treatment position of left‐side breast radiotherapy using anatomical features extracted from supine free breathing CT scans with multiple machine learning models and outweighed the important features that affect the optimal positioning prediction. The challenge to improve predictive models for left‐breast treatment positioning remains open. Specifically, the availability of strong features is always the key to constructing better predictive models. For ongoing work, we are applying for clinical trials to produce more experimental data and improving the predictive models by utilizing powerful feature extraction techniques, such as Convolutional Neural Networks and atlas‐based organ segmentation.

## CONFLICT OF INTEREST

The authors have no relevant conflicts of interest to disclose.
